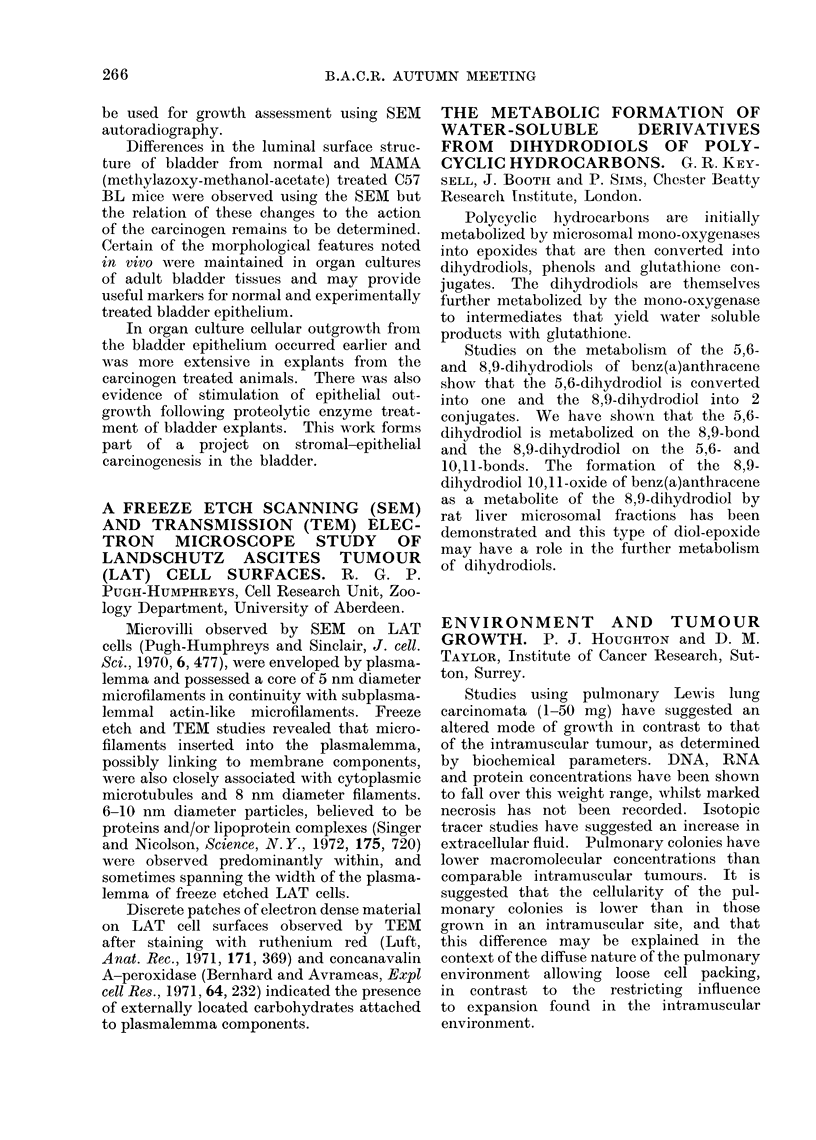# Proceedings: A freeze etch scanning (SEM) and transmission (TEM) electron microscopic study of Landschutz ascites tumour (LAT) cell surfaces.

**DOI:** 10.1038/bjc.1975.56

**Published:** 1975-02

**Authors:** R. G. Pugh-Humphreys


**A FREEZE ETCH SCANNING (SEM) AND TRANSMISSION (TEM) ELECTRON MICROSCOPE STUDY OF LANDSCHUTZ ASCITES TUMOUR (LAT) CELL SURFACES.** R. G. P. PUGH-HUMPHREYS, Cell Research Unit, Zoology Department, University of Aberdeen.

Microvilli observed by SEM on LAT cells (Pugh-Humphreys and Sinclair, *J. cell. Sci.*, 1970, **6**, 477), were enveloped by plasmalemma and possessed a core of 5 nm diameter microfilaments in continuity with subplasmalemmal actin-like microfilaments. Freeze etch and TEM studies revealed that microfilaments inserted into the plasmalemma, possibly linking to membrane components, were also closely associated with cytoplasmic microtubules and 8 nm diameter filaments. 6–10 nm diameter particles, believed to be proteins and/or lipoprotein complexes (Singer and Nicolson, *Science, N.Y.*, 1972, **175**, 720) were observed predominantly within, and sometimes spanning the width of the plasmalemma of freeze etched LAT cells.

Discrete patches of electron dense material on LAT cell surfaces observed by TEM after staining with ruthenium red (Luft, *Anat. Rec.*, 1971, **171**, 369) and concanavalin A–peroxidase (Bernhard and Avrameas, *Expl cell Res.*, 1971, **64**, 232) indicated the presence of externally located carbohydrates attached to plasmalemma components.